# I-FABP as Biomarker for the Early Diagnosis of Acute Mesenteric Ischemia and Resultant Lung Injury

**DOI:** 10.1371/journal.pone.0115242

**Published:** 2014-12-26

**Authors:** Rachel G. Khadaroo, Spyridon Fortis, Saad Y. Salim, Catherine Streutker, Thomas A. Churchill, Haibo Zhang

**Affiliations:** 1 Department of Surgery, University of Alberta, Edmonton, Alberta, Canada; 2 Division of Critical Care Medicine, University of Alberta, Edmonton, Alberta, Canada; 3 Departments of Anesthesia, Medicine and Physiology, Keenan Research Center for Biomedical Science of St. Michael's Hospital and University of Toronto, Toronto, Ontario, Canada; 4 Department of Pathology, St. Michael's Hospital, Toronto, Ontario, Canada; University of Pecs Medical School, Hungary

## Abstract

Acute mesenteric ischemia (AMI) is a life-threatening condition that can result in multiple organ injury and death. A timely diagnosis and treatment would have a significant impact on the morbidity and mortality in high-risk patient population. The purpose of this study was to investigate if intestinal fatty acid binding protein (I-FABP) and α-defensins can be used as biomarkers for early AMI and resultant lung injury. C57BL/6 mice were subjected to intestinal ischemia by occlusion of the superior mesenteric artery. A time course of intestinal ischemia from 0.5 to 3 h was performed and followed by reperfusion for 2 h. Additional mice were treated with N-acetyl-cysteine (NAC) at 300 mg/kg given intraperitoneally prior to reperfusion. AMI resulted in severe intestinal injury characterized by neutrophil infiltrate, myeloperoxidase (MPO) levels, cytokine/chemokine levels, and tissue histopathology. Pathologic signs of ischemia were evident at 1 h, and by 3 h of ischemia, the full thickness of the intestine mucosa had areas of coagulative necrosis. It was noted that the levels of α-defensins in intestinal tissue peaked at 1 h and I-FABP in plasma peaked at 3 h after AMI. Intestinal ischemia also resulted in lung injury in a time-dependent manner. Pretreatment with NAC decreased the levels of intestinal α-defensins and plasma I-FABP, as well as lung MPO and cytokines. In summary, the concentrations of intestinal α-defensins and plasma I-FABP predicted intestinal ischemia prior to pathological evidence of ischemia and I-FABP directly correlated with resultant lung injury. The antioxidant NAC reduced intestinal and lung injury induced by AMI, suggesting a role for oxidants in the mechanism for distant organ injury. I-FABP and α-defensins are promising biomarkers, and may guide the treatment with antioxidant in early intestinal and distal organ injury.

## Introduction

Acute mesenteric ischemia (AMI) can occur through many mechanisms. There are occlusive causes such as arterial embolus or thrombosis, or venous thrombosis as well as non-occlusive causes such as non-occlusive mesenteric infarction (NOMI) secondary to low flow states. The gastrointestinal tract forms an important defensive barrier. Its unique vascular supply and predisposition towards both ischemia and mucosal hypoxia put it at high risk for injury. As the result of an injury, the gastrointestinal tract becomes a major pathogenic source of bacteria and inflammatory mediators that subsequently lead to a septic response. The mortality rates in patients with AMI have not changed since the 1940's, with rates ranging from 60–80% [1]–[3]. As such, finding novel biomarkers that can predict gastrointestinal injury prior to pathological changes is needed.

An important factor for the high mortality rate of AMI is a failure of a timely diagnosis, since there is no simple marker for diagnosing intestinal injury [4], [5]. In particular, the signs and symptoms of intestinal ischemia are vague and non-specific [6]. The hallmark symptoms of abdominal pain and tenderness are lost when patients are critically ill since they are often unconscious, intubated, and/or sedated. Conventional markers of intestinal ischemia, serum lactate level and white cell count (WBC), lack sensitivity and specificity, whereas invasive diagnostic tests, such as computer tomography (CT) or angiography, expose patients to risk of contrast-induced nephropathy[7].

Because of the challenges of diagnosis, the estimation of the mortality attributable to AMI is very difficult. This is best illustrated by a British post-mortem review where more than 50% of deaths due to AMI were not diagnosed until the time of autopsy[8]. A more timely diagnosis of gastrointestinal injury would allow for an earlier intervention, and be beneficial in many ways, such as: 1) avoidance of surgery by earlier aggressive fluid resuscitation and/or medical correction of the diagnosis, 2) less post-operative morbidity and improved survival if surgery was indicated, 3) provide a surrogate marker to the risk of further systemic injury, and 4) ultimately aid in guiding therapy towards preventing distant organ injury.

The intestine epithelia have unique cell types. The disruption of intestinal epithelial cell integrity can release factors that can be measured and quantified. Paneth cells are specialized epithelial cells that were first described over a century ago as granulated cells at the base of the small intestinal crypts of Lieberkuhn [9]. There are approximately 5 to 12 Paneth cells in each small intestinal crypt. These specialized secretory epithelial cells express high levels of human α-defensins (HD) 5 and 6 [10]. Upon intestinal damage and exposure to heat killed or live bacteria or microbial products, such as LPS, Paneth cells release their granules resulting in increased concentration of HD into the intestinal lumen [11]. In addition, intestinal epithelial cells express a family of 15 kDa cytoplasmic fatty acid binding proteins (I-FABP). I-FABP is predominantly expressed in the villi and may provide a useful marker for differentiating early intestinal ischemia [12], [13].

As such, we hypothesize that intestinal derived factors can be used as biomarkers for early diagnosis of AMI. Using a model of intestinal ischemia/reperfusion, we found that intestinal and lung injury correlated with inflammation. We also found that intestinal α-defensins and plasma I-FABP were detectable prior to intestinal histologic injury. Our murine study corroborates that I-FABP can be used as a biomarker for AMI [14], [15].

## Materials and Methods

### Animals

All animal experimental protocols were approved by the Animal Care and Use Committee at the St. Michael's Hospital. Recommendations in the Guide for the Care and Use of Laboratory Animals were strictly followed. The animals were kept in a pathogen-free condition with 12:12 h light∶dark cycle and fed *ad libitum* on standard murine diet and tap water. Throughout the surgical procedure, the animals were maintained under general anaesthesia while being placed on a heating pad to maintain a constant body temperature at 37°C.

### Animal Model of Acute Intestinal Ischemia

C57BL/6 male mice (8–10 wk old, 25–28 g; Jackson Laboratories, Bar Harbor, ME) were maintained under anesthesia throughout the entire duration of the study by intraperitoneal (IP) injection of ketamine (200 mg/kg; Ketalean, Bimeda-MTC Animal Health, Cambridge, ON) and xylazine (10 mg/kg; Rompun, Bayer, Agriculture Division, Animal Health, Etobicoke, ON). Using hind-limb pinch method, and visually monitoring respiratory rate, the depth of anesthesia was monitored every 5 minutes and maintained with additional dose if anesthesia depth changed. A tracheotomy was performed and a sterile angiocatheter (Angiocath, 20 gauge, Becton Dickinson Infusion Therapy Systems, Sandy, UT) and was inserted into the trachea. Mice were subjected to intestinal ischemia by occlusion of the superior mesenteric artery (SMA) with a microvascular clip. Sham animals were subjected to the same surgical interventions and time course without SMA occlusion. This model simulates not only the clinical setting of NOMI as reperfusion occurs during the resuscitation phase, but also the occlusive intestinal ischemia that is followed by intervention resulting in reperfusion of the ischemic injury. A time course of intestinal ischemia lasting 0.5, 1, 2, and 3 h was followed by reperfusion for 2 h. The animals were under anesthesia throughout the entire experimental procedure including reperfusion. After 2 h of reperfusion, mice were euthanized and blood sample collected through the inferior vena cava and organs harvested for assessment. Only animals that survived to the endpoint of the study were used for analysis.

To study the effects of oxidative stress, we injected IP an antioxidant, N-acetyl-cysteine (NAC) at 300 mg/kg IP. Animals were pretreated animals prior to AMI and reperfusion. Control animals were give vehicle solution of normal saline (No-NAC).

### Intestinal Histology

Ileal tissue samples were fixed in buffered 10% formalin solution (BDH, Toronto, ON) at room temperature for one week. Fixed intestinal segments were paraffinised and sectioned onto slides. Slides were stained with H&E and were assessed by a pathologist in a blinded fashion. Intestinal injury was graded on a 6-point histopathological grading (see Table 1). An average number was obtained in ten high powered field (hpf) observations.

**Table:** **Table 1.** Intestinal injury histopathologic grading.

Grade	Injury
0	Normal villi
1	Capillary congestion
2	Moderate lifting of epithelial layer
3	Massive epithelial lifting
4	Denuded villi, dilated capilliaries
5	Digestion & disintegration of lamina propria, hemorrhage, & ulceration

Intestinal injury was graded by both a 6-point mucosal injury score and the average infiltration of polymorphonuclear (PMN) leukocytes quantified in 10 high powered fields (hpf).

### Lung Histology

At the end of the experiments, a segment of the lungs were collected for histology. The lungs were fixed by immersion in 10% buffered neutral formalin (BDH, Toronto, ON) and processed using standard histological techniques. There were 8 different pathological features that were examined, including alveolar edema/exudates, hemorrhage, and interstitial/alveolar cellular infiltration. They were scored on a scale of 1–3 (0, absent; 1, mild; 2, moderate; 3, severe) from six fields randomly chosen per slide by a pathologist in a blinded fashion. Polymorphonuclear (PMN) infiltrates were quantified in ten high powered fields (hpf) for each slide.

### Myeloperoxidase (MPO) Assay

Intestinal and lung tissue segments were homogenized and processed in 800 µl of an equivalent volume of 50 mmol/L phosphate-buffered saline (pH 6.0) containing 0.5% hexadecyltrimethylammonium bromide. Tissue segments were sonicated at 40 W for 1 minute. Samples were centrifuged at 21,000x*g* for 20 min at 4°C and supernatants were collected. The protein content of all the samples was determined using the Pierce BCA Protein Assay (Pierce, Rockford, IL). MPO activity was assessed by adding 25 µL of 3 mM H_2_O_2_ as previously described [16]. Change in absorbance was measured using a Cobas FARA II centrifuge analyzer (Roche Diagnostic Systems, Montclair, NJ) at A655 nm over 3 minutes duration. MPO activity was quantified and data displayed as Units/mg of total protein.

### I-FABP ELISA

The concentration of I-FABP in the plasma was determined using ELISA and by following manufacturer's instructions (Hycult Biotechnology, Uden, the Netherlands). 50 µL of plasma from each sample was used for the measurements. Data was presented as concentration of I-FABP in plasma in ng/ml.

### Inflammatory Cytokines/Chemokine ELISA

Inflammatory cytokines and chemokines were measured from lung tissue homogenates and plasma. Tumor necrosis factor-alpha (TNF-α), monocyte chemoattractant protein-1 (MCP-1), keratinocyte-derived cytokine (KC), and interleukin-1β (IL-1β) were measured using a mouse Cytometric Bead Array (CBA) inflammation kit (BD Bioscience, Mississauga, ON). The protocol was followed according to manufacturer's instructions.

### Intestinal α-defensins ELISA

The Paneth cells in mice express a functional homologue of human α-defensins-5 and -6. In fact, there is 60% (for α-defensin-5) and 55% (for α-defensin-6) similarity in protein structures between mouse and human α-defensins [17]–[21]. As such, anti-human antibodies were used successfully to detect for murine intestine α-defensins. The 96-well plates were coated overnight with mouse anti-human Human Neutrophil Peptide(HNP)-1-3 monoclonal antibody (HyCult Biotechnology, Uden, The Netherlands) which cross reacts with human defensin (HD) 5 and 6. After thorough washing and blocking non-specific protein binding with BSA, HNP standards and samples were added to the wells and incubated for 1 h. The samples were incubated for 1 h with 1∶1,000 rabbit anti-human HNP-1-3 polyclonal antibody (Host Defence Research Centre, Toronto, ON), followed by incubation for 1 h with 1∶4,000 peroxidase-conjugated goat anti-rabbit IgG (Jackson ImmunoResearch). The 3,3′,5,5′-tetramethyl-benzidine substrate (Sigma) was added and the reaction was stopped by using 1 M sulphuric acid. The absorbance was read at 450 nm. Data was presented as ng/mL normalised to mg of total protein.

### Statistical Analysis

Statistical analysis was performed using Prism 5.0 for Windows (GraphPad Software Inc., San Diego, CA). Results were expressed as mean ± standard error mean. Comparison between the groups was performed using a two-way analysis of variance (ANOVA). A *p*-value <0.05 was considered as significant. If significant (*p<0.05*), the Tukey post-hoc test was used to examine differences between groups.

## Results

### Survival and Intestinal Injury following Acute Mesenteric Ischemia (AMI)

**Figure 1:**
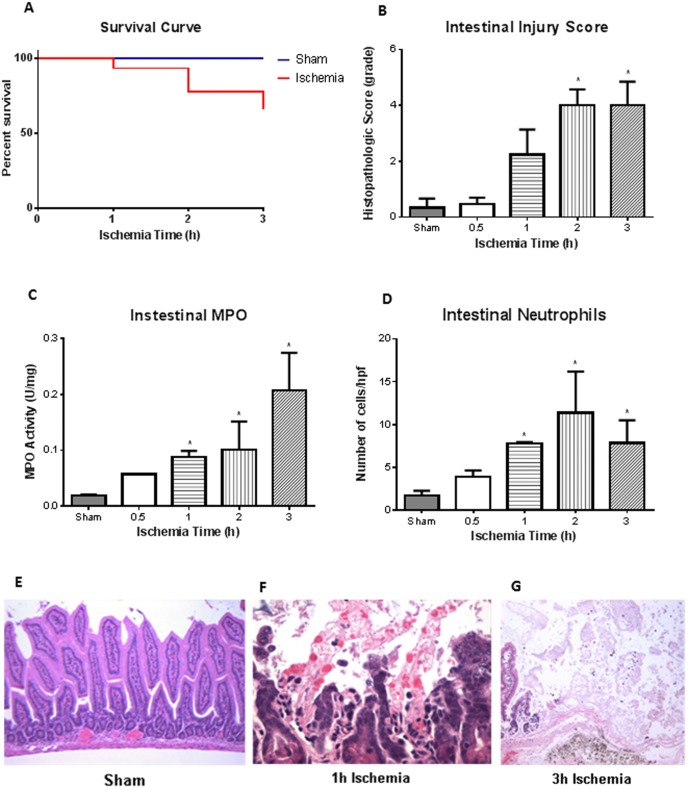
Survival and intestinal injury following acute mesenteric ischemia and reperfusion. ***A***, There was no statistical difference in mice survival following 0.5, 1, 2, or 3 h (n = 6, 12, 15, 5, respectively) ischemia times with 2 h of reperfusion compared to sham treated mice (n = 5). ***B***, Histological examination of intestinal tissue illustrated that there was no difference between 0.5 h ischemia and sham treated mice; however, tissue injury started to become visible after 1 h ischemia with significance in histological score seen after 2 and 3 h of ischemia (n = 3 for all groups). ***C***, Intestinal myeloperoxidase (MPO) corroborated histological score with increased MPO activity found after 1, 2, and 3 h ischemia compared to sham mice(n = 3 for all groups). ***D***, MPO activity mirrored the number of neutrophils found infiltrating the intestinal tissue, where compared to sham treated mice, significant neutrophil infiltrate was found after 1, 2, and 3 h ischemia (n = 3 for all groups). ***E***, Hematoxylin and eosin staining illustrated that intestinal tissue in sham treated animals was normal (100x). ***F***, After 1 h ischemia, however, we begin to observe denuded villi and increased polymorphonuclear (PMN) infiltrates (400x). ***G***, At 3 h ischemia, the full thickness of the intestinal mucosa had areas of coagulative necrosis and complete mucosal erosions (400x). * *p<0.05* compared to sham treated mice.

C57BL/6 mice were subjected to intestinal ischemia at different time points: 0.5, 1, 2 and 3 hours (n = 6, 12, 15, 5, respectively). This was followed by reperfusion for 2 h. Sham animals (n = 5) were not exposed to the complete occlusion of the superior mesenteric artery but were left open for the full duration of the procedure. The mice were given fluid resuscitation, at 0.5 ml/h intraperitoneally (IP), resulting in 93% survival after 1 h, 77% survival at 2 h, and 66% survival at 3 h of ischemia (Fig. 1A). There was no significant difference in survival between sham and mice exposed to ischemia at any time point (Chi square test). There was, an increase in intestinal injury corresponding with increasing ischemia times, as noted by histological score (Fig. 1B). Histology images showed sham animals had normal epithelia (Fig. 1E), which was similar to the 0.5 h ischemia mice (image not shown) (n = 3 for all groups). However, there were pathological signs of ischemia with epithelial erosion and increased polymorphonuclear (PMN) infiltrates after 1 h of ischemia (Fig. 1F). After 3 h of ischemia, the full thickness of the intestinal mucosa had areas of coagulative necrosis and complete mucosal erosions (Fig. 1G). Increased intestinal histological score corresponded to increased intestinal myeloperoxidase (MPO) activity (Fig. 1C). There were significant differences in MPO activity after 1, 2, and 3 h of ischemia animals compared to sham controls. There was also increased number of neutrophils that infiltrated the intestinal mucosa at 1, 2, and 3 h of ischemia (Fig. 1D).

### Conventional vs. Novel Biomarkers used for Diagnosis of AMI

**Figure 2:**
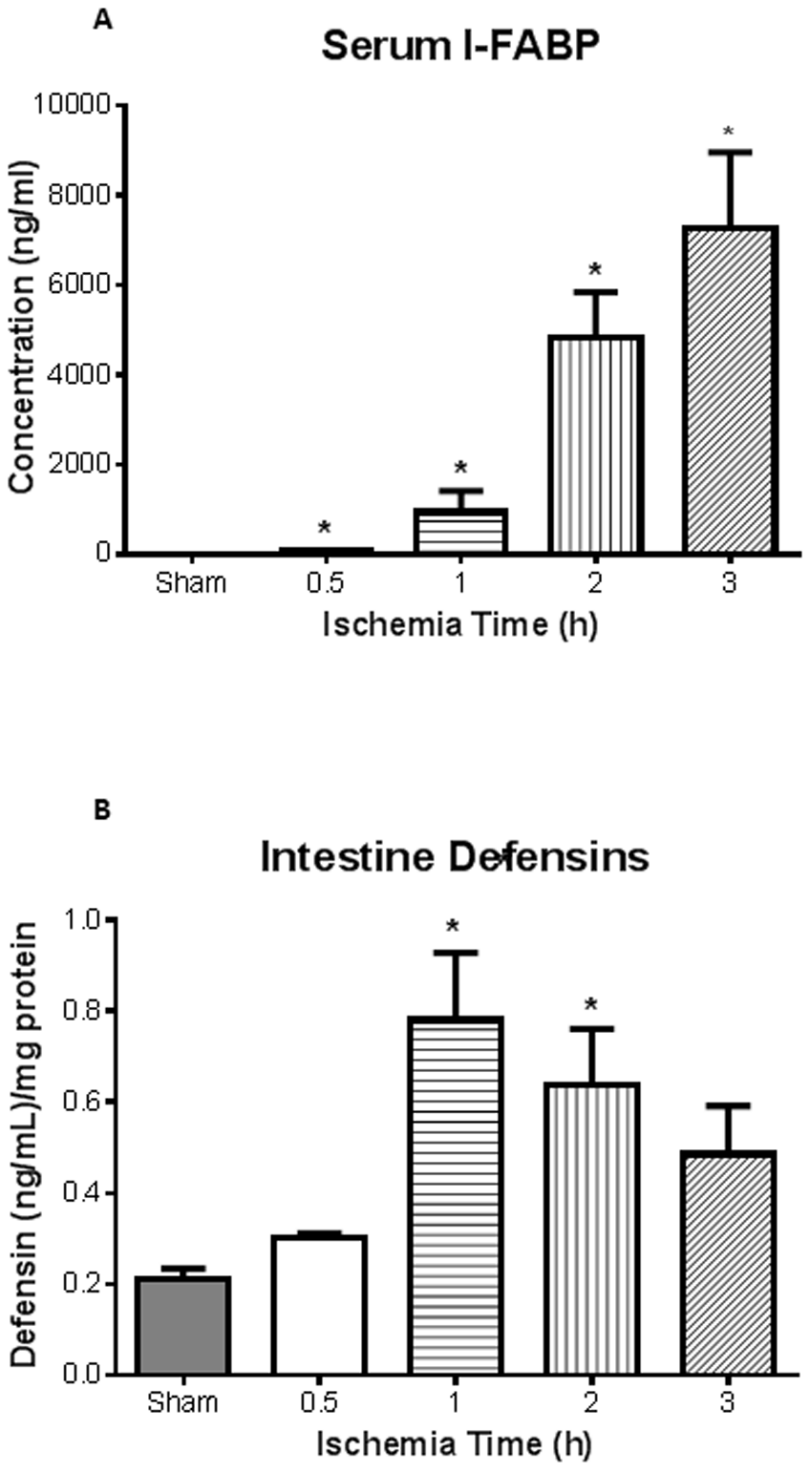
I-FABP as novel biomarker for ischemia/reperfusion injury. ***A***, Plasma intestinal (I)-FABP, measured via ELISA, was found to be significantly elevated after ischemia at all time points compared to sham animals (n = 3, 4, 4, 4, 5 for sham, 0.5, 1, 2 and 3 h, respectively). Of note was the detection of I-FABP after 0.5 h (n = 3), well before pathological injury was observed (64±18 versus 6.9±0.9 for shams). ***B***, Detection of defensins in intestinal homogenates illustrated that significant levels after 1 and 2 h ischemia compared to shams (n = 3 for all groups). * *p<0.05* compared to sham treated mice.

Levels of lactate and white blood cells (WBC), conventional markers used clinically, showed no statistical difference between sham and AMI at all time points of intestinal ischemia (data not shown).Plasma intestinal (I)-FABP levels directly correlated with ischemia times with significant increase seen after 2 and 3 h of intestinal ischemia (Fig. 2A; n = 3, 4, 4, 4, 5 for sham, 0.5, 1, 2 and 3 h). I-FABP was significantly elevated following 0.5 h of ischemia which was even prior to pathological changes. The plasma measurements of I-FABP also directly correlated with lung injury (R^2^ = 0.88, p = 0.037). The levels of intestinal homogenate α-defensins (HD 5 and 6) measured by ELISA showed a significant increase at 1 h and 2 h of intestinal ischemia (Fig. 2B).

### Distant Organ Injury – Increased Lung Inflammation and Injury

**Figure 3:**
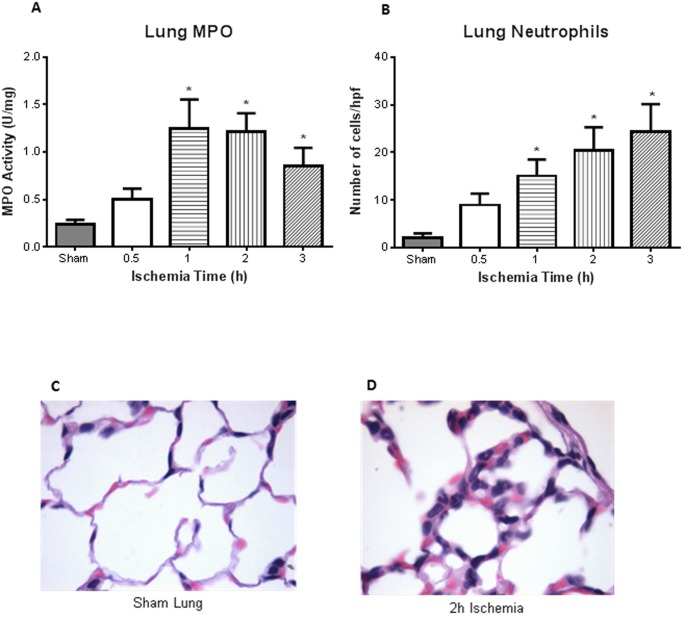
Distant organ injury – increased lung MPO and injury. ***A***, MPO on lung homogenates illustrated increased activity after 1, 2, and 3 h intestinal ischemia compared to sham treated mice (n = 4 for all groups). ***B***, Quantification of the number of neutrophils infiltrate in the lungs showed significance also after 1, 2, and 3 h of ischemia (n = 4, 5, 6, 6, 7 for sham, 0.5, 1, 2, 3 respectively). ***C***, Histology of a sham treated mice illustrated normal lung parenchyma (400x). ***D***, However, PMN infiltrates were observed in the lung after 2 h of intestinal ischemia (400x). * *p<0.05* compared to sham treated mice.

**Figure 4:**
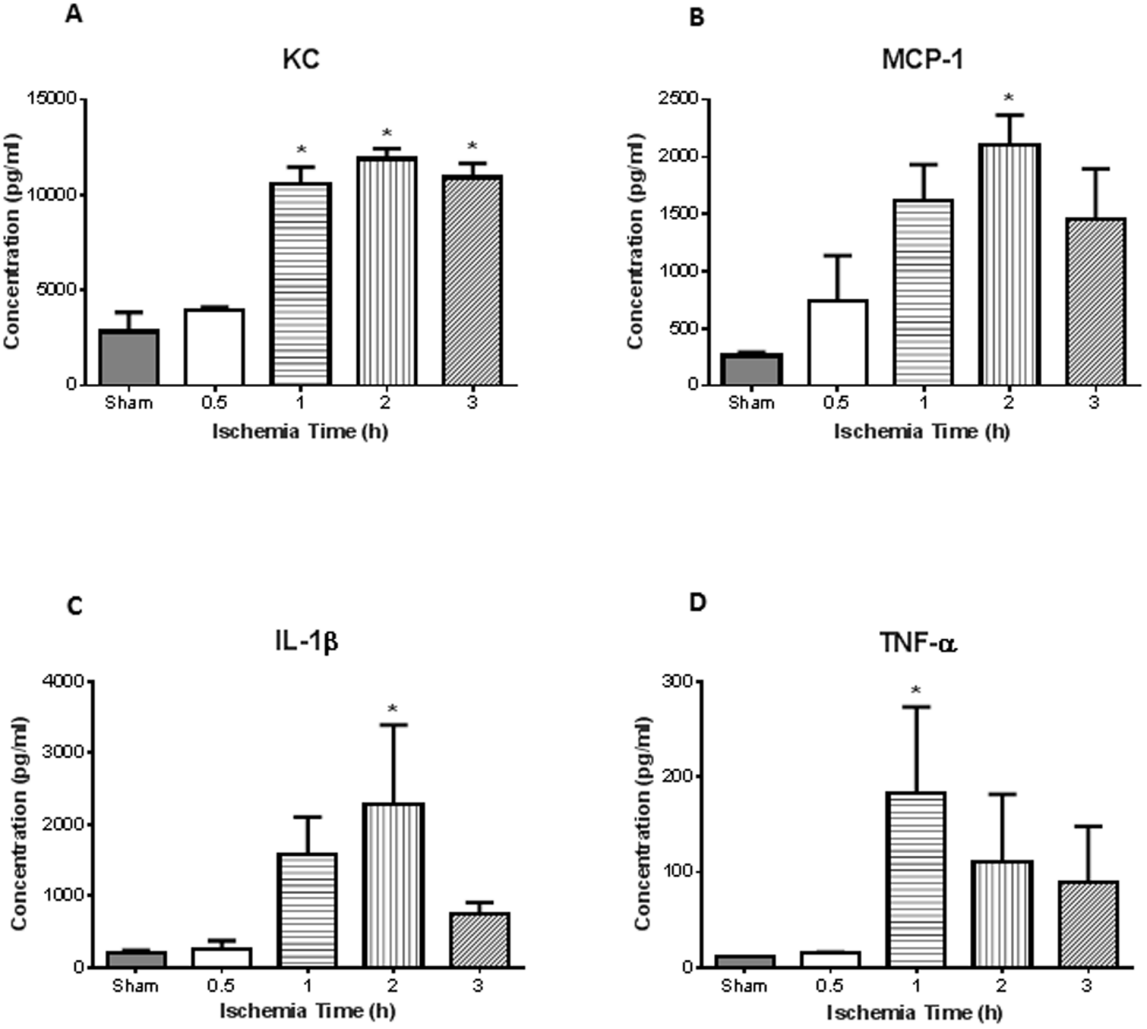
Increased lung inflammation following intestinal ischemia/reperfusion. Lung homogenates were used to measure inflammatory chemokines and cytokines. ***A***, There was greater concentration of keratinocyte-derived chemokine (KC) after 1, 2, and 3 h of intestinal ischemia compared to sham controls. ***B***, Meanwhile monocyte chemoattractant protein-1 (MCP-1) showed significance after 2 h. ***C***, Inflammatory cytokine IL-1β was found significantly elevated at 2 h ischemia, while, ***D***, TNF-α was significantly elevated after 1 h of intestinal ischemia. (n = 3 for sham, n = 4 for all ischemia treated mice) * *p<0.05* compared to sham treated mice.

Intestinal ischemia resulted in evidence of lung injury in a time-dependent manner. The neutrophil sequestration in the lung, as reflected by MPO activity of lung homogenates, directly correlated with the duration of intestinal ischemia. Mean lung MPO was 0.24 U/mg at 0.5 h of intestinal ischemia and significantly increased to >1.25 U/mg at 1 h of ischemia (p<0.05) (Fig. 3A; n = 4 for all groups). Histopathological examination of the lung showed normal appearing lung parenchyma in the sham animals (Fig. 3C). However, PMN leukocyte infiltrates were observed after 2 h intestinal ischemia (Fig. 3D). Similar to the intestine, there was increased neutrophil infiltrate, with significance seen after 1, 2, and 3 h of intestinal ischemia (Fig. 3B; n = 4, 5, 6, 6, 7 for sham, 0.5, 1, 2, 3). Lung homogenate corroborated histological findings in that we observed significant increase in neutrophil chemokine KC after 1, 2, and 3 h of intestinal ischemia (Fig. 4A; n = 3 for sham, n = 4 for all ischemia treated mice). There was also increased monocyte chemokine MCP-1 at 2 h of ischemia (Fig. 4B). Of the cytokines measured in the lung homogenates, we found increased levels of IL-1β and TNF-α at 2 h and 1 h after intestinal ischemia, respectively (Fig. 4C and D).

### Reactive Oxygen Stress Causes Distant Organ Injury

**Figure 5:**
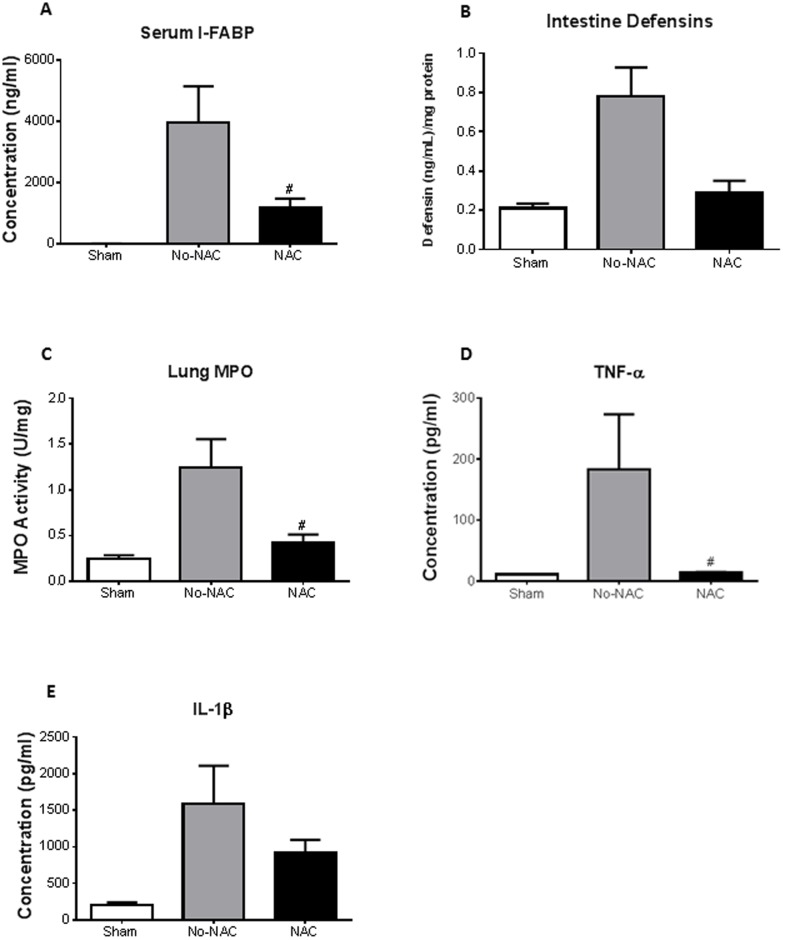
NAC reduces oxidative injury. To determine the mechanism behind distal organ injury, mice were pretreated intraperitoneally with, N-acetyl-cysteine (NAC). ***A***, Compared to mice given vehicle control (No-NAC), NAC treated mice had significantly lower plasma I-FABP after 2 h of intestinal ischemia. ***B***, NAC also affected defensins at 1 h ischemia, though no significance was achieved. ***C***, MPO activity of lung homogenates illustrated a significant reduction in mice that were treated with NAC compared to vehicle controls (No-NAC) at 1 h ischemia. ***D***, There was also significant reduction in TNF-α cytokine in lung homogenates after NAC treatment at 1 h ischemia. ***E***, At 2 h ischemia, there was slight reduction in IL-1β in the NAC treated mice compared to vehicle controls, though significance was not achieved probably due to the short half-life of NAC. (n = 3 for shams, n = 4 for both No-NAC and NAC) ^#^ *p<0.05* compared to vehicle (No-NAC) treated mice.

We hypothesized that oxidative stress might be the mechanism through which acute mesenteric ischemia and reperfusion causes intestinal and distant organ injury. To test our hypothesis, we used a potent antioxidant, N-acetyl-cysteine (NAC), given intraperitoneally to the animals prior to ischemia/reperfusion (n = 3 for shams, n = 4 for both No-NAC and NAC). NAC pretreatment significantly lowered plasma I-FABP levels at 2 h ischemia compared to vehicle-treated control (no-NAC) (Fig. 5A). NAC also had an effect on intestine α-defensins concentration (Fig. 5B). There was a significant reduction in lung MPO levels at 1 h (Fig. 5C) as well as TNF-α at 1 h of intestinal ischemia (Fig. 5D) with NAC treatment compared to no-NAC. The effect of NAC became less significant at 2 h and 3 h of ischemia. NAC treatment had the tendency in reducing IL-1β in lung homogenates (Fig. 5E) after 2 h ischemia.

## Discussion

AMI is a life-threatening intra-abdominal emergency where the outcome depends on rapid diagnosis. AMI can be caused by occlusive or non-occlusive mechanisms. In both cases, rapid vascular resuscitation results in intestinal reperfusion. The intestinal ischemia/reperfusion induces mucosal barrier damage which results in the translocation of micro-organisms and endotoxins from the gut lumen into the body's inner tissues [22]–[25], in combination with the production of oxygen-derived free radicals[26], [27] and other intestinally derived factors[28], [29], as well as pro-inflammatory cytokines [30]. A self-perpetuating signaling cascade that has the potential to escalate into a vicious cycle of continuously increasing intestinal permeability, translocation of microbes and stronger inflammatory response develops. All of these factors ultimately contribute to the onset of systemic inflammatory response syndrome (SIRS), sepsis, and multiple organ dysfunction syndrome (MODS). Of these organ injuries, acute lung injury (ALI) and acute respiratory distress syndrome (ARDS) are a common cause of morbidity and mortality following AMI [31], [32]. In fact, the mortality due to ALI has remained at 44% for the past two decades [33]; highlighting the need for a novel biomarker to allow for earlier detection.

In contrast to other organs (i.e. heart, pancreas, and liver), intestinal ischemia has been more difficult to diagnosis by serologic makers. This difficulty could be due to an overlap of intestinal biomarkers with other systemic markers of injury, such as lactate, and/or hepatic metabolism that clears biological markers that have drained through the portal vein. Despite these challenges, there are unique cell types found within the intestinal epithelia, and molecules that are released upon disruptions of epithelial integrity can be measured and quantified. Here we hypothesized that intestinal fatty acid binding protein (I-FABP) and human α-defensins (HD) released from damaged intestinal epithelia can be used as novel biomarkers and are more accurate than traditional markers (WBC and lactate) in the diagnosis of intestinal ischemia. We found that I-FABP was significantly elevated after 0.5 hours of ischemia, much earlier than any visible intestinal pathology. Importantly, plasma measurements of I-FABP were directly correlated with local intestinal as well as distant lung injury. Congruently, α-defensins were elevated in intestinal homogenates prior to histopathological observations. This also provides a relevant tissue biomarker that could be used when endoscopic biopsies are performed to asses for intestinal pathology including ischemia [34]. This is particularly important since a recent study showed that 15% of patients who had features of ischemia on CT scan had normal endoscopy [35]. Thus, early pathology may be better identified by using a blood and/or tissue biomarkers.

By using a potent antioxidant, N-acetyl-cysteine (NAC), we illustrated that plasma levels of I-FABP and tissue intestinal α-defensins and pro-inflammatory cytokines can bedimished, thus identifying oxidative stress as a possible mechanism for intestinal injury. Since NAC is a safe and effective treatment for acetaminophen toxicity [36], our study provides a rationale to further investigate its use for the medical treatment of intestinal ischemia.

I-FABP is a low molecular mass cytoplasmic protein. It is found specifically in the epithelial cells of the small intestine. Upon intestinal injury and/or mucosal barrier disruptions, due to poor mesenteric blood flow and necrosis, I-FABP protein is rapidly released into the circulatory system. The idea to use I-FABP as a biomarker for mesenteric ischemia has been around since the 1990s [37], [38]. However, the test is still not available in clinical practice for several potential reasons: the need for larger prospective studies in humans to validate I-FABP for clinical use, lower clinical profile of AMI, and ultimately the need for the creation of a rapid bedside test to improve the clinical utility. Our results corroborate previous clinical studies examining the role of I-FABP in the diagnosis of intestinal ischemia [38]–[41]. The largest study included 52 patients with small bowel ischemia. It demonstrated that I-FABP had a good sensitivity for intestinal ischemia however it failed to exceed the specificity of creatinine phosphokinase or lactate dehydrogenase [39]. Though other studies have shown I-FABP as a potentially useful biomarker for identifying intestinal ischemia [41], [42], none of the studies have examined the role of I-FABP in predicting distant organ injury.

In our animal model, we have found that AMI results in both direct intestinal injury and acute lung injury. At the initial ischemic phase, decreased mesenteric blood flow causes anoxic cellular injury, activation of hydrolases, loss of cellular membrane's selective permeability, and ultimately cellular death [43]. The reperfusion phase of AMI further aggravates tissue damage and is considered to be the main driver of local as well as distant inflammation and tissue injury [44]. Immune cells infiltrate the intestine upon resupply of blood and initiate inflammatory responses. The compromised mucosal barrier results in microbial translocation that further exacerbates the inflammatory processes. We found that with increasing ischemia times, the greater the histopathology of local and distant organ. This was consistent with the number of neutrophil infiltrates and reactive oxygen species present in homogenates. To further examine the role of oxidants in the pathophysiology, especially in distant organ injury, N-acetyl-cysteine (NAC) was given to the animals prior to ischemia. This prevented lung injury as demonstrated by decreased lung neutrophil influx and inflammatory cytokine production. In addition, antioxidant treatment also reversed the elevation of the biomarkers I-FABP and α-defensins. This observation suggests that use of antioxidant as a possible treatment modality in early intestinal and distal organ injury due to AMI. Our study demonstrates that I-FABP as a promising biomarker for AMI, and can potentially predict resulting ALI.

The major limitation of this study is the small sample size and although our animal model produces common clinical features of AMI, the mechanisms by which AMI induces lung injury remain to be elucidated. The use of I-FABP and α-defensins as biomarkers needs to be validated in critically ill patients and those who present emergently with abdominal emergencies.

## Conclusions

AMI broadly affects critically ill patients in the areas of trauma, transplantation, cardiac surgery, shock, and sepsis. I-FABP and α-defensins predicted intestinal ischemia prior to pathological evidence of ischemia in our animal model of AMI. I-FABP also directly correlated with resultant lung injury. Further clinical studies are required to confirm the role of I-FABP and α-defensins as biomarkers for AMI. Pretreatment of AMI with the antioxidant NAC reduced lung injury, suggesting a role for oxidants in this mechanism and a potential treatment modality. Early diagnosis of intestinal ischemia resulting in timely treatment can decrease morbidity and mortality in critically ill patients.

## References

[1] Klempnauer J, Grothues F, Bektas H, Pichlmayr R (1997) Long-term results after surgery for acute mesenteric ischemia. Surgery 121:239–243. 9068664 10.1016/s0039-6060(97)90351-2

[2] Wilcox MG, Howard TJ, Plaskon LA, Unthank JL, Madura JA (1995) Current theories of pathogenesis and treatment of nonocclusive mesenteric ischemia. Dig Dis Sci 40:709–716. 7720458 10.1007/BF02064966

[3] Kassahun WT, Schulz T, Richter O, Hauss J (2008) Unchanged high mortality rates from acute occlusive intestinal ischemia: six year review. Langenbecks Arch Surg 393:163–171. 18172675 10.1007/s00423-007-0263-5

[4] Khadaroo RG, Marshall JC (2008) Gastrointestinal dysfunction in the critically ill: can we measure it? Crit Care 12:180. 18828891 10.1186/cc7001 PMC2592736

[5] Evennett NJ, Petrov MS, Mittal A, Windsor JA (2009) Systematic review and pooled estimates for the diagnostic accuracy of serological markers for intestinal ischemia. World J Surg 33:1374–1383. 19424744 10.1007/s00268-009-0074-7

[6] Howard TJ, Plaskon LA, Wiebke EA, Wilcox MG, Madura JA (1996) Nonocclusive mesenteric ischemia remains a diagnostic dilemma. Am J Surg 171:405–408. 8604831 10.1016/S0002-9610(97)89619-5

[7] Glenister KM, Corke CF (2004) Infarcted intestine: a diagnostic void. ANZ J Surg 74:260–265. 15043738 10.1111/j.1445-2197.2004.02956.x

[8] Wilson C, Gupta R, Gilmour DG, Imrie CW (1987) Acute superior mesenteric ischaemia. Br J Surg 74:279–281. 3580801 10.1002/bjs.1800740417

[9] Clevers HC, Bevins CL (2013) Paneth cells: maestros of the small intestinal crypts. Annu Rev Physiol 75:289–311. 23398152 10.1146/annurev-physiol-030212-183744

[10] Cunliffe RN, Rose FR, Keyte J, Abberley L, Chan WC, et al (2001) Human defensin 5 is stored in precursor form in normal Paneth cells and is expressed by some villous epithelial cells and by metaplastic Paneth cells in the colon in inflammatory bowel disease. Gut 48:176–185. 11156637 10.1136/gut.48.2.176 PMC1728187

[11] Ayabe T, Satchell DP, Wilson CL, Parks WC, Selsted ME, et al (2000) Secretion of microbicidal alpha-defensins by intestinal Paneth cells in response to bacteria. Nat Immunol 1:113–118. 11248802 10.1038/77783

[12] Sacchettini JC, Hauft SM, Van Camp SL, Cistola DP, Gordon JI (1990) Developmental and structural studies of an intracellular lipid binding protein expressed in the ileal epithelium. J Biol Chem 265:19199–19207. 1699943

[13] Ockner RK, Manning JA (1974) Fatty acid-binding protein in small intestine. Identification, isolation, and evidence for its role in cellular fatty acid transport. J Clin Invest 54:326–338. 4211161 10.1172/JCI107768 PMC301560

[14] Cesar Machado MC, Barbeiro HV, da Silva FP, de Souza HP (2012) Circulating fatty acid binding protein as a marker of intestinal failure in septic patients. Crit Care 16:455. 23130611 10.1186/cc11653 PMC3672560

[15] Derikx JP, Poeze M, van Bijnen AA, Buurman WA, Heineman E (2007) Evidence for intestinal and liver epithelial cell injury in the early phase of sepsis. Shock 28:544–548. 17607153 10.1097/shk.0b013e3180644e32

[16] Davreux CJ, Soric I, Nathens AB, Watson RW, McGilvray ID, et al (1997) N-acetyl cysteine attenuates acute lung injury in the rat. Shock 8:432–438. 9421857

[17] Eisenhauer PB, Harwig SS, Lehrer RI (1992) Cryptdins: antimicrobial defensins of the murine small intestine. Infect Immun 60:3556–3565. 1500163 10.1128/iai.60.9.3556-3565.1992 PMC257361

[18] Jones DE, Bevins CL (1992) Paneth cells of the human small intestine express an antimicrobial peptide gene. J Biol Chem 267:23216–23225. 1429669

[19] Jones DE, Bevins CL (1993) Defensin-6 mRNA in human Paneth cells: implications for antimicrobial peptides in host defense of the human bowel. FEBS Lett 315:187–192. 8417977 10.1016/0014-5793(93)81160-2

[20] Ouellette AJ, Lualdi JC (1990) A novel mouse gene family coding for cationic, cysteine-rich peptides. Regulation in small intestine and cells of myeloid origin. J Biol Chem 265:9831–9837. 2351676

[21] Selsted ME, Miller SI, Henschen AH, Ouellette AJ (1992) Enteric defensins: antibiotic peptide components of intestinal host defense. J Cell Biol 118:929–936. 1500431 10.1083/jcb.118.4.929 PMC2289569

[22] Deitch EA (2002) Bacterial translocation or lymphatic drainage of toxic products from the gut: what is important in human beings? Surgery 131:241–244. 11894026 10.1067/msy.2002.116408

[23] Sheng ZY, Dong YL, Wang XH (1991) Bacterial translocation and multiple system organ failure in bowel ischemia and reperfusion. Chin Med J (Engl) 104:897–903. 1800029

[24] Marshall JC, Christou NV, Meakins JL (1993) The gastrointestinal tract. The "undrained abscess" of multiple organ failure. Ann Surg 218:111–119. 8342990 10.1097/00000658-199308000-00001 PMC1242919

[25] Pastores SM, Katz DP, Kvetan V (1996) Splanchnic ischemia and gut mucosal injury in sepsis and the multiple organ dysfunction syndrome. Am J Gastroenterol 91:1697–1710. 8792684

[26] Granger DN (1988) Role of xanthine oxidase and granulocytes in ischemia-reperfusion injury. Am J Physiol 255:H1269–1275. 3059826 10.1152/ajpheart.1988.255.6.H1269

[27] McKelvey TG, Hollwarth ME, Granger DN, Engerson TD, Landler U, et al (1988) Mechanisms of conversion of xanthine dehydrogenase to xanthine oxidase in ischemic rat liver and kidney. Am J Physiol 254:G753–760. 3163235 10.1152/ajpgi.1988.254.5.G753

[28] Dayal SD, Hauser CJ, Feketeova E, Fekete Z, Adams JM, et al (2002) Shock mesenteric lymph-induced rat polymorphonuclear neutrophil activation and endothelial cell injury is mediated by aqueous factors. J Trauma 52:1048–1055 discussion 1055. 12045629 10.1097/00005373-200206000-00005

[29] Adams JM, Hauser CJ, Adams CA Jr, Xu DZ, Livingston DH, et al (2001) Entry of gut lymph into the circulation primes rat neutrophil respiratory burst in hemorrhagic shock. Crit Care Med 29:2194–2198. 11700422 10.1097/00003246-200111000-00023

[30] Deitch EA, Xu D, Franko L, Ayala A, Chaudry IH (1994) Evidence favoring the role of the gut as a cytokine-generating organ in rats subjected to hemorrhagic shock. Shock 1:141–145. 7749933 10.1097/00024382-199402000-00010

[31] Harward TR, Brooks DL, Flynn TC, Seeger JM (1993) Multiple organ dysfunction after mesenteric artery revascularization. J Vasc Surg 18:459–467 discussion 467–459. 8377240 10.1067/mva.1993.48586

[32] Vincent JL, Akca S, De Mendonca A, Haji-Michael P, Sprung C, et al (2002) The epidemiology of acute respiratory failure in critically ill patients(*). Chest 121:1602–1609. 12006450 10.1378/chest.121.5.1602

[33] Phua J, Badia JR, Adhikari NK, Friedrich JO, Fowler RA, et al (2009) Has mortality from acute respiratory distress syndrome decreased over time?: A systematic review. Am J Respir Crit Care Med 179:220–227. 19011152 10.1164/rccm.200805-722OC

[34] Lozano-Maya M, Ponferrada-Diaz A, Gonzalez-Asanza C, Nogales-Rincon O, Senent-Sanchez C, et al (2010) Usefulness of colonoscopy in ischemic colitis. Rev Esp Enferm Dig 102:478–483. 20670068 10.4321/s1130-01082010000800004

[35] Al-Khowaiter SS, Brahmania M, Kim E, Madden M, Harris A, et al (2014) Clinical and endoscopic significance of bowel-wall thickening reported on abdominal computed tomographies in symptomatic patients with no history of gastrointestinal disease. Can Assoc Radiol J 65:67–70. 23142403 10.1016/j.carj.2012.01.002

[36] Whyte AJ, Kehrl T, Brooks DE, Katz KD, Sokolowski D (2010) Safety and effectiveness of acetadote for acetaminophen toxicity. J Emerg Med 39:607–611. 19022608 10.1016/j.jemermed.2008.05.007

[37] Kanda T, Fujii H, Fujita M, Sakai Y, Ono T, et al (1995) Intestinal fatty acid binding protein is available for diagnosis of intestinal ischaemia: immunochemical analysis of two patients with ischaemic intestinal diseases. Gut 36:788–791. 7797132 10.1136/gut.36.5.788 PMC1382688

[38] Lieberman JM, Sacchettini J, Marks C, Marks WH (1997) Human intestinal fatty acid binding protein: report of an assay with studies in normal volunteers and intestinal ischemia. Surgery 121:335–342. 9068676 10.1016/s0039-6060(97)90363-9

[39] Kanda T, Tsukahara A, Ueki K, Sakai Y, Tani T, et al (2011) Diagnosis of ischemic small bowel disease by measurement of serum intestinal fatty acid-binding protein in patients with acute abdomen: a multicenter, observer-blinded validation study. J Gastroenterol 46:492–500. 21298292 10.1007/s00535-011-0373-2

[40] Cronk DR, Houseworth TP, Cuadrado DG, Herbert GS, McNutt PM, et al (2006) Intestinal fatty acid binding protein (I-FABP) for the detection of strangulated mechanical small bowel obstruction. Curr Surg 63:322–325. 16971202 10.1016/j.cursur.2006.05.006

[41] Thuijls G, van Wijck K, Grootjans J, Derikx JP, van Bijnen AA, et al (2011) Early diagnosis of intestinal ischemia using urinary and plasma fatty acid binding proteins. Ann Surg 253:303–308. 21245670 10.1097/SLA.0b013e318207a767

[42] Acosta S, Nilsson T (2012) Current status on plasma biomarkers for acute mesenteric ischemia. J Thromb Thrombolysis 33:355–361. 22081293 10.1007/s11239-011-0660-z

[43] de Groot H, Rauen U (2007) Ischemia-reperfusion injury: processes in pathogenetic networks: a review. Transplant Proc 39:481–484. 17362763 10.1016/j.transproceed.2006.12.012

[44] Grootjans J, Lenaerts K, Derikx JP, Matthijsen RA, de Bruine AP, et al (2010) Human intestinal ischemia-reperfusion-induced inflammation characterized: experiences from a new translational model. Am J Pathol 176:2283–2291. 20348235 10.2353/ajpath.2010.091069 PMC2861093

